# Supplementary material for: Standalone bio-interventional uveoscleral outflow enhancement for intraocular pressure reduction in open-angle glaucoma: One-year results from a prospective multicenter real-world evidence study (NCT05506423)
Source: PLoS One. 2026 Jun 26;21(6):e0351552. doi: 10.1371/journal.pone.0351552 (PMC13308803; doi:10.1371/journal.pone.0351552)
Supplement: S4 File — (PDF) [file pone.0351552.s004.pdf]

IANTREK, INC.

PROTOCOL ITR-CYC-041
VERSION 5

AN OBSERVATIONAL REGISTRY STUDY OF SAFETY AND EFFECTIVENESS OUTCOMES THROUGH 24 MONTHS POSTOPERATIVELY FOLLOWING CYCLOPEN™ MICRO-INTERVENTIONAL CYCLODIALYSIS SYSTEM PROCEDURES IN PATIENTS WITH OPEN ANGLE GLAUCOMA (THE CREST STUDY)

SPONSOR:

IANTREK, INC
151 E. POST RD, SUITE 111
WHITE PLAINS, NY 10601

PHONE: [redacted]

TABLE OF CONTENTS

LIST OF ABBREVIATIONS 4

STUDY SYNOPSIS 5

1. INTRODUCTION 10
   1.1. Physiology of Aqueous Outflow and Surgical Treatment Options for IOP Lowering 10
   1.2. Surgical Cyclodialysis 11

2. STUDY OBJECTIVES 12
   2.1. Effectiveness Outcomes 12
   2.2. Safety Outcomes 13

3. STUDY DESIGN 13

4. STUDY POPULATION 13
   4.1. Inclusion Criteria 13
   4.2. Exclusion Criteria 13

5. STUDY INTERVENTION 14

6. PARTICIPANT DISCONTINUATION/WITHDRAWAL 14
   6.1. Termination 14
   6.2. Completion 15

7. STUDY DATA COLLECTION 15
   7.1. Preoperative Clinical Assessments 15
   7.2. Surgical Procedure Data 16
   7.3. Postoperative Clinical Assessments 16

8. ADVERSE EVENTS 17
   8.1. Adverse Device Effects 17
   8.2. Serious Adverse Events 18
      8.2.1. Serious Ocular Adverse Events 18
   8.3. Adverse Event Assessment 18
   8.4. Severity 19
   8.5. Relationship to Study Device 19
      8.5.1. Expectedness 19
   8.6. Adverse Event Documentation and Reporting 21
      8.6.1. Expedited Adverse Event Reporting 21

9. STATISTICAL CONSIDERATIONS 22
9.1. Sample Size 22
9.2. Analysis Populations 22
9.2.1. Safety Population 22
9.2.2. Treated-Eye Population 22
9.3. Data Analysis Conventions 23
9.4. Baseline Definition 23
9.5. Descriptive Statistics 23
9.6. Effectiveness Outcome Analysis 23
9.6.1. Exploratory Analyses 24
9.7. Safety Outcome Analysis 24
9.7.1. Visual Field Change 24
9.7.2. Endothelial Cell Loss 24
9.8. Multiplicity and Missing Data 24

10. SUPPORTING DOCUMENTATION AND OPERATIONAL CONSIDERATIONS 25
10.1. Data Management 25
10.2. Data Capture, Entry, and Quality Control 25
10.3. Data Archiving 25
10.4. Records Retention 25
10.5. Quality Assurance 25
10.6. Protocol Amendments 25
10.7. Publication Policy 26

11. ETHICAL AND REGULATORY CONSIDERATIONS 26
11.1. Risks and Benefits of Participation 26
11.2. Patient Information and Informed Consent 26
11.3. Participant Confidentiality 27
11.4. Institutional Review 27

12. REFERENCES 28

13. INVESTIGATOR ATTESTATION 30

LIST OF ABBREVIATIONS

Abbreviation	Term
ADE	Adverse device effect
AE	Adverse event
BCDVA	Best-corrected distance visual acuity
C:D	Cup-to-disc ratio
CDVA	Corrected distance visual acuity
CFR	Code of (United States) Federal Regulations
EC	Ethics committee
eCRF	Electronic case report form
EDC	Electronic data capture
FDA	Food and Drug Administration (United States)
GEE	Generalized estimating equation
HIPAA	Health Insurance Portability and Accountability Act
ICH GCP	International Council for Harmonization on Good Clinical Practice
ICF	Informed consent form
IOL	Intraocular lens
IOP	Intraocular pressure
IRB	Institutional Review Board
mmHg	Millimeters of mercury
MD	Mean deviation
Nd:YAG	neodymium-doped yttrium aluminum garnet laser
OAG	Open angle glaucoma
OVD	Ophthalmic viscosurgical device
PDF	Portable document format
SAE	Serious adverse event
UADE	Unanticipated adverse device effect
UCDVA	Uncorrected distance visual acuity

NOTE: The first occurrence of some abbreviations is not spelled out in the document (e.g., units of measure).

STUDY SYNOPSIS

**Sponsor:**	Iantrek, Inc.<br>151 E. Post Road, Suite 111<br>White Plains, NY 10601<br>Phone: [redacted]
**Title:**	An Observational Registry Study of Safety and Effectiveness Outcomes through 24 Months Postoperatively Following CycloPen™ Micro-Interventional Cyclodialysis System Procedures in Patients with Open Angle Glaucoma (The CREST Study)
**Objective:**	To evaluate postoperative outcomes in a real-world setting after use of the CycloPen Micro-Interventional Cyclodialysis System (CycloPen System) in patients with Open Angle Glaucoma (OAG), as characterized by:<br>• Change in intraocular pressure (IOP)<br>• Use of ocular hypotensive medications<br>• Ocular adverse events (AE)
**Study Population:**	The CREST Registry consecutively enrolls consenting adults for whom IOP-lowering surgery using the CycloPen System was attempted or completed. Surgeries may be either in combination with cataract surgery or standalone procedures (*participants may be treatment-naïve or have had previous glaucoma surgery)*.
**Study Design:**	Multicenter, observational study in which all eligible consenting participants in the CREST Registry with OAG who underwent IOP-lowering ophthalmic surgery with the CycloPen System are enrolled.<br><br>Data will be retrospectively collected from participants' preoperative ophthalmic examination(s) and the surgical procedure in which the CycloPen System was used. Data from specified postoperative visits will generally be collected prospectively; however, if specified postoperative visit(s) occurred prior to patient consent for participation, data from these visits will be collected retrospectively.
**Statistical Considerations:**	Due to additional IOP reduction that may be achieved with cataract surgery, effectiveness will be assessed separately for the 2 cohorts below:<br>• Cohort 1: CycloPen procedure combined with cataract extraction/intraocular lens (IOL) implantation, and<br>• Cohort 2: CycloPen procedure as a standalone surgical intervention<br><br>For exploratory analyses, these 2 cohorts may be further sub-divided, depending on the nature of IOP-lowering interventions performed prior to, or in conjunction with, the CycloPen surgical procedure. Safety will be

	assessed in the pooled cohort and in each cohort separately.
**Description of Enrolling Sites/Facilities:**	Participants will be enrolled by ophthalmologists performing glaucoma surgical procedures at ambulatory surgery centers or hospitals in the United States and Latin America.
**Phase:**	Post-market
**Estimated Study Duration:**	56 months (32 months' enrollment + 24 months' participant follow-up)
**Effectiveness Outcomes:**	Effectiveness outcomes, listed in order of clinical importance, are defined as follows:<br><br>• Mean change in 12-month postoperative IOP in comparison with baseline<br>• Mean percent IOP reduction at 12 months postoperatively in comparison with baseline, while using the same or fewer ocular hypotensive medications without additional IOP-lowering surgical or laser procedures<br>• Mean change in number of ocular hypotensive medications used at 12 months postoperatively in comparison with baseline<br>• Mean change in 24-month postoperative IOP in comparison with baseline<br>• Mean percent IOP reduction at 24 months postoperatively in comparison with baseline, while using the same or fewer ocular hypotensive medications without additional IOP-lowering surgical or laser procedures<br>• Mean change in number of ocular hypotensive medications used at 24 months postoperatively in comparison with baseline<br>• Proportion of eyes achieving 12-month postoperative IOP ≥ 20% lower than baseline, while using the same or fewer ocular hypotensive medications without additional IOP-lowering surgical or laser procedures<br>• Proportion of eyes achieving 24-month postoperative IOP ≥ 20% lower than baseline, while using the same or fewer ocular hypotensive medications without additional IOP-lowering surgical or laser procedures<br>• Proportion of eyes achieving 12- and 24-month postoperative IOP ≥ 20% lower than baseline without use of ocular hypotensive medication or IOP-lowering surgical or laser procedures<br>• Proportion of eyes achieving 12- and 24-month postoperative IOP ≥ 6 mmHg and ≤ 18 mmHg, while using the same or fewer ocular

	hypotensive medications without additional IOP-lowering surgical or laser procedures<br>• Proportion of eyes achieving 12- and 24-month postoperative IOP ≥ 6 mmHg and ≤ 18 mmHg, without use of ocular hypotensive medication or IOP-lowering surgical or laser procedures<br>• For eyes with baseline IOP > 21 mmHg, proportion of eyes achieving 12- and 24-month postoperative IOP ≤ 21 mmHg and ≥ 20% lower than baseline, while using the same or fewer ocular hypotensive medications without additional IOP-lowering surgical procedures
**Safety Outcomes:**	• Incidence of ocular AEs<br>• Secondary surgical interventions for IOP control<br>• Clinically significant ocular findings observed > 30 days postoperatively that are not considered to be AEs<br>• Postoperative corrected distance visual acuity (CDVA)

Schedule of Visits/Assessments:

Participants will be approached for enrollment after IOP-lowering surgery using the CycloPen System.

Data will be collected from the preoperative visit(s) that directly preceded surgery, the surgical procedure, and visits occurring at approximately 1, 6, 12, 18, and 24 months postoperatively, as shown in ***Figure 1: Participant Evaluation Flow Chart***. Data will also be collected from interim postoperative visits associated with procedure-related adverse events and/or concomitant procedures, as well as changes in ocular hypotensive medication use. Data from the preoperative visit and the surgical procedure will be collected retrospectively. Data from specified postoperative visits will generally be collected prospectively; however, if specified postoperative visit(s) occurred prior to patient consent for participation, data from these visits will be collected retrospectively.

As this study uses data from an observational registry, examinations at each visit are performed per the investigator's standard of care. Specific data to be collected from each visit, if the relevant examination is performed, is presented in ***Table 1: Clinical Assessment and Procedure Data to be Collected by Evaluation Time Period.***

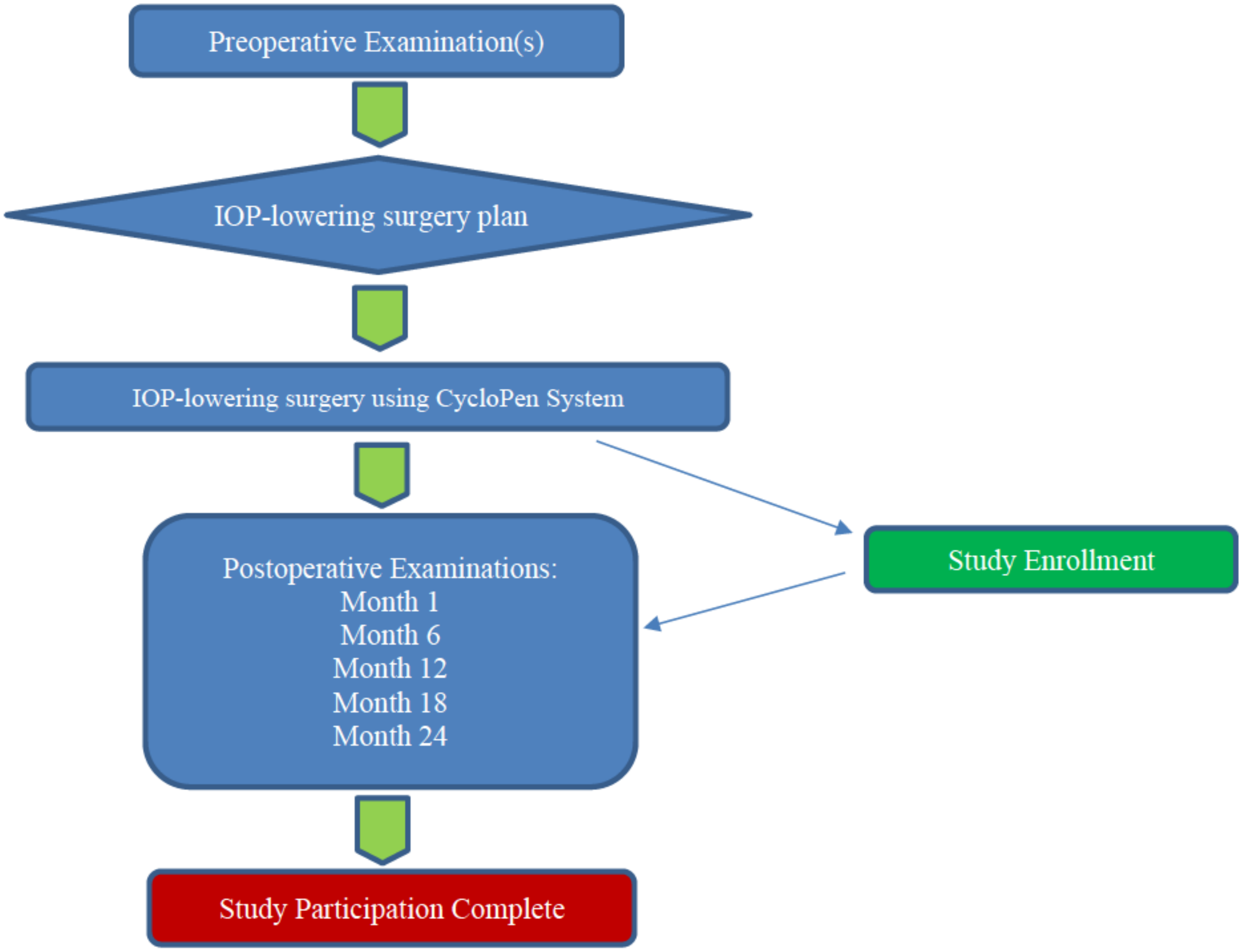

Figure 1: Participant Evaluation Flow Chart

Table 1: Clinical Assessment and Procedure Data to be Collected by Evaluation Time Period (Assessments and Procedures performed are per investigator's standard practice)

Assessment/Procedure	Preoperative[1] (Day -90 to -1)	Surgery (Day 0)	Postoperative Evaluations 1 Month (Days 15 – 45)	6 Months (Days 150 – 210)	12 Months (Days 275-455)	18 Months (Days 457-637)	24 Months (Days 640 –820)
Demographic information	X						
Medical history	X						
Ocular medical history	X						
Ocular hypotensive medication use	X		X	X	X	X	X
Biometry (axial length & keratometry)[8]	X						
Target postoperative refraction[5]	X						
UCDVA	X						
CDVA & associated refraction	X		X	X	X	X	X
Slit lamp exam	X		X	X	X	X	X
Gonioscopy[2]	X	X[4]	X	X	X	X	X
IOP	X		X	X	X	X	X
Dilated fundus exam[7,9]	X				X		X
Automated perimetry MD[9]	X[6]				X		X
Specular microscopy[3]	X[6]				X		X
Surgical procedure data		X					
Adverse event assessment		X	X	X	X	X	X

[1] If assessments were performed at multiple preoperative visits, data from the assessment closest to the date of surgery will be collected.
[2] Gonioscopy imaging may be collected, if available.
[3] If performed as part of investigator's standard practice; central endothelial cell counts and images may be collected, if available. Central endothelial cell counts and images taken in the immediate postoperative period should be collected if no preoperative data is available. Images will be assessed for reliability, then associated endothelial cell counts will be summarized with descriptive statistics.
[4] If performed during surgical procedure.
[5] This data should be collected if a combined cataract/IOL surgical procedure was performed.
[6] Preoperative MD score and specular microscopy data collected may be within 6 months prior to surgery.
[7] If macular OCT performed in conjunction with the fundus examination, imaging may be collected.
[8] Keratometry data is not required for eyes in Cohort 2 (standalone surgery).
[9] The dilated fundus exam and visual field perimetric assessments performed at intervals outside of the 12 month and 24 month visit windows should be documented on an Unscheduled Visit eCRF.

1. INTRODUCTION

As described in the American Academy of Ophthalmology Preferred Practice Pattern, open-angle glaucoma (OAG) represents a significant public health problem. In the United States, OAG (not including the major forms of secondary open-angle glaucoma, pseudoexfoliation glaucoma and pigmentary glaucoma) affects an estimated 3.3 million people[1], and glaucoma of all types is one of the leading causes of legal blindness.[2,3,4]

Glaucoma is typically associated with an abnormal elevation of intraocular pressure (IOP), and management of glaucoma involves reduction of IOP to a range deemed safe by the treating physician. Intraocular pressure reduction may be achieved with use of ocular hypotensive medication and/or laser, filtering, or cyclodestructive surgery. Results from randomized controlled trials and other studies reinforce this expectation and provide evidence that the more IOP is lowered, the more likely it is that progressive loss of vision will be halted.[5-18]

When IOP is uncontrolled, progressive loss of vision from optic neuropathy can result, which is permanent and irreversible. When beginning therapy, the treating physician assumes that the measured pretreatment pressure range has contributed to optic nerve damage and is likely to cause additional damage in the future. To prevent additional damage, an initial target IOP is established that is generally ≥ 20% lower than the pretreatment IOP, depending on the clinical findings. Further reduction of the target IOP may be justified by the severity of existing optic nerve damage, the level of the measured pretreatment IOP, the rapidity with which the damage occurred, and other risk factors. In general, the more advanced the damage, the lower the initial target IOP should be. During 7 years of follow-up, patients from the Advanced Glaucoma Intervention Study whose IOP was consistently <18 mmHg had minimal visual field progression as compared with patients who had higher IOP. [19]

1.1. Physiology of Aqueous Outflow and Surgical Treatment Options for IOP Lowering

In both the normal and the glaucomatous eye, aqueous fluid is produced by the secretory epithelium of the ciliary body, which extends circumferentially around the interior of the eye posterior to the iris, covering the ciliary processes. Aqueous circulates anteriorly through the pupil and exits the eye by means of two independent pathways: (1) the trabecular meshwork, and (2) uveoscleral outflow.

With aqueous outflow via the trabecular meshwork (known as pressure dependent outflow), aqueous passes through the trabecular meshwork into Schlemm's canal, then into aqueous veins that drain into the episcleral venous plexus (the pressure in these veins is generally around 10 mmHg).

With uveoscleral outflow (known as pressure independent outflow), aqueous moves from the anterior chamber through the intermuscular spaces of the ciliary muscle into the supraciliary and suprachoroidal space, then out the eye through the sclera or the perivascular spaces of the emissarial channels (perforation in the sclera for the passage of nerves and blood vessels).[20] The

rate of uveoscleral outflow in humans is about 0.2-0.5 microliters/minute, while the rate of aqueous production is about 2-3 microliters per minute. Aqueous flows from the anterior chamber into the supraciliary and suprachoroidal spaces because of this pressure gradient. In monkeys, the posterior suprachoroidal pressure is 4 mmHg lower than the anterior chamber pressure. This negative pressure is due to the high oncotic absorptive potential of uveal vessels.[21]

Medical and surgical therapies for treatment of elevated IOP are directed towards reducing aqueous production, increasing aqueous outflow through the normal outflow channels, or a combination of both. Surgical interventions are directed almost exclusively at increasing outflow, either internally, by creating an alternate or improved pathway to Schlemm's canal or the suprachoroidal space/uveoscleral outflow, or externally, by directing aqueous outflow to alternative drainage sites outside of the interior of the eye to the sub-tenon and subconjunctival space.

1.2. Surgical Cyclodialysis

Experience from the pharmacologic treatment of glaucoma with prostaglandin analogues demonstrates best-in-class IOP lowering effect for drugs targeting uveoscleral outflow. Decreased uveoscleral outflow resistance has been previously studied in the presence of cyclodialysis clefts, which separate the ciliary body from the scleral spur, thus creating a free communication channel between the anterior chamber and the supraciliary space. In the presence of a cyclodialysis cleft, uveoscleral outflow is markedly enhanced.[22]

While cyclodialysis has been evaluated as a minimally invasive IOP-lowering surgical procedure, the challenges associated with procedural success relate to: (1) the ability to control the size of the cyclodialysis (a cyclodialysis too large may result in hypotony leading to ciliochoroidal detachment) and (2) prevention of spontaneous cleft closure, which may lead to a sudden rise in IOP.

The CycloPen Micro-Interventional Cyclodialysis System (CycloPen System) is a manual surgical instrument set used for the *ab-interno* construction or modification of a cyclodialysis cleft via controlled dissection of a plane in the supraciliary space. After creation of the cleft, the CycloPen may be used to deliver viscous material and/or minimally-manipulated allograft scleral tissue to reinforce and maintain the cleft, thus reducing the likelihood of spontaneous cleft closure. Cleft size may vary depending on the IOP-lowering goal for the patient and surgeon preference; larger clefts may require positioning of allograft tissue at each edge of the cleft, and/or application of a larger amount of ophthalmic viscoelastic, while smaller clefts may be adequately reinforced using a single allograft and/or less viscoelastic.

2. STUDY OBJECTIVES

The purpose of this study is to evaluate postoperative outcomes in a real-world setting after IOP-lowering surgical procedures using the CycloPen System are attempted or completed in patients with OAG.

2.1. Effectiveness Outcomes

Effectiveness outcomes, listed in order of clinical importance, are defined as follows:

1. Mean change in 12-month postoperative IOP in comparison with baseline
2. Mean percent IOP reduction at 12 months postoperatively in comparison with baseline, while using the same or fewer ocular hypotensive medications without additional IOP-lowering surgical or laser procedures
3. Mean change in number of ocular hypotensive medications used at 12 months postoperatively in comparison with baseline
4. Mean change in 24-month postoperative IOP in comparison with baseline
5. Mean percent IOP reduction at 24 months postoperatively in comparison with baseline, while using the same or fewer ocular hypotensive medications without additional IOP-lowering surgical or laser procedures
6. Mean change in number of ocular hypotensive medications used at 24 months postoperatively in comparison with baseline
7. Proportion of eyes achieving 12-month postoperative IOP ≥ 20% lower than baseline, while using the same or fewer ocular hypotensive medications without additional IOP-lowering surgical or laser procedures
8. Proportion of eyes achieving 24-month postoperative IOP ≥ 20% lower than baseline, while using the same or fewer ocular hypotensive medications without additional IOP-lowering surgical or laser procedures
9. Proportion of eyes achieving 12- and 24-month postoperative IOP ≥ 20% lower than baseline without use of ocular hypotensive medication or IOP-lowering surgical or laser procedures
10. Proportion of eyes achieving 12- and 24-month postoperative IOP ≥ 6 mmHg and ≤ 18 mmHg, while using the same or fewer ocular hypotensive medications without additional IOP-lowering surgical or laser procedures
11. Proportion of eyes achieving 12- and 24-month postoperative IOP ≥ 6 mmHg and ≤ 18 mmHg, without use of ocular hypotensive medication or IOP-lowering surgical or laser procedures
12. For eyes with baseline IOP > 21 mmHg, proportion of eyes achieving 12- and 24-month postoperative IOP ≤ 21 mmHg and ≥ 20% lower than baseline, while using the same or fewer ocular hypotensive medications without additional IOP-lowering surgical procedures

2.2. Safety Outcomes

- Incidence of ocular AEs
- Secondary surgical interventions for IOP control
- Clinically significant ocular findings observed > 30 days postoperatively that are not considered to be AEs
- Postoperative CDVA

3. STUDY DESIGN

This is a multicenter, observational registry study in which eligible consenting participants for whom CycloPen System IOP-lowering ophthalmic surgery (attempted or completed) are consecutively enrolled. Data from the preoperative visit and the surgical procedure will be collected retrospectively. Data from specified postoperative visits will generally be collected prospectively; however, if specified postoperative visit(s) occurred prior to patient consent for participation, data from these visits will be collected retrospectively.

4. STUDY POPULATION

The study will enroll consenting adults with OAG in whom IOP-lowering surgery using the CycloPen System was attempted or completed. *Note: IOP-lowering surgery using the CycloPen System, which was "attempted, but not completed" is defined as surgery in which neither a viscous nor non-viscous material was delivered to the cyclodialysis cleft.*

Surgeries may have been performed in combination with cataract surgery or as standalone procedures. Participants may have been treatment-naïve or had previous glaucoma surgery.

4.1. Inclusion Criteria

Participants must meet the criteria below to be enrolled in the study:

- Diagnosis of OAG
- Underwent a CycloPen System surgical procedure for the purpose of lowering IOP
- Provide signed written consent using the IRB/EC-approved study-specific ICF
- Ability, comprehension, and willingness to follow study instructions

4.2. Exclusion Criteria

There are no exclusionary criteria for study participation.

4.3. Study Cohorts

Due to the additional IOP reduction that may be achieved with cataract surgery, effectiveness will be assessed separately for each of the 2 cohorts below:

- Cohort 1: CycloPen procedure combined with cataract extraction/intraocular lens (IOL) implantation, and
- Cohort 2: CycloPen procedure as a standalone surgical intervention.

  Within Cohort 2, eyes will be further classified as “refractory” or “non-refractory”. Eyes with refractory glaucoma meet at least one of the following criteria:

  - Failed ≥ 1 incisional intraocular glaucoma filtering surgeries (*Note: This criterion does not include failure with MIGs devices)*
  - Failed ≥ 1 cilioablative procedures (e.g., cryotherapy, cyclodiode therapy)
  - Have a condition (e.g., conjunctival scarring, uveitis) in which conventional incisional glaucoma surgery like trabeculectomy would be more likely to fail than for an eye with uncomplicated primary OAG.

  Any eye that does not meet the refractory criteria will be considered to have non-refractory glaucoma.

5. STUDY INTERVENTION

The CycloPen Cyclodialysis System is a manual surgical instrument set for the *ab-interno* construction or modification, reinforcement, and repair of a cyclodialysis cleft via controlled surgical dissection in the supraciliary plane and delivery of viscous or non-viscous materials for structural maintenance, reinforcement, and durability of the cleft.

This instrument system is registered with the United States Food and Drug Association (FDA) as a Class I exempt manual ophthalmic cannula.

6. PARTICIPANT DISCONTINUATION/WITHDRAWAL

6.1. Termination

Participants may be terminated from the study due to:

- Voluntary withdrawal of consent
- Health issues that preclude ongoing follow-up
- Adverse event (AE) related to use of the CycloPen System during attempted but not completed surgical procedure (*Note: Relevant clinical data should be collected regarding the AE(s) until resolution or stabilization of the event for participants terminated for this reason)*
- Geographic relocation
- Loss to follow-up

- Other administrative reasons (e.g., registry termination by Iantrek, non-adherence with registry requirements)

Iantrek should be promptly notified of a participant's early termination, and information regarding the termination should be documented in the source records and on the relevant study electronic case report form (eCRF).

Study site personnel should take the following steps to minimize the likelihood of early termination during the study:

- During the initial postoperative visit, thoroughly evaluate the participant for potential health or motivational issues or other life circumstances that may negatively affect the ability to return for scheduled postoperative examinations; emphasize to the participant the importance of returning for these examinations.
- Attempt to follow-up with participants who do not return for scheduled examination visits. *Note: A minimum of 3 documented attempts via telephone, email, or regular mail should be made to contact participants who do not return for scheduled visits. If there is no reply, the site should consider sending a letter by certified mail (with request for notification of receipt of delivery). Participants who are non-responsive to these follow-up attempts will be considered lost to follow-up.*

6.2. Completion

Participants will have completed study participation if they did not terminate prior to completion of their 24-month postoperative examination.

7. STUDY DATA COLLECTION

To evaluate participant outcomes associated with use of the CycloPen System, data will be retrospectively collected from the preoperative visit(s) that directly preceded surgery, and the surgical procedure. Data from specified postoperative visits occurring at approximately 1, 6, 12, 18, and 24 months after surgery will generally be collected prospectively; however, if specified postoperative visit(s) occurred prior to patient consent for participation, data from these visits will be collected retrospectively.

Data collection activities by visit and the visit window range for each visit are summarized in ***Table 1: Clinical Assessment and Procedure Data to be Collected by Evaluation Time Period***.

7.1. Preoperative Clinical Assessments

Where available, the data listed below will be collected from assessments performed ≤ 90 days prior to surgery. If assessments were performed multiple times, the assessment that is temporally closest to the date of surgery will be collected:

- Demographic information
- Relevant non-ocular medical history

- Ocular medical history
- Hypotensive medications used (ocular and systemic)
- Uncorrected distance visual acuity (UCDVA)
- Corrected distance visual acuity (CDVA) with refraction
- Slit lamp examination
- Gonioscopy
- Intraocular Pressure (IOP)
- Dilated fundus examination
- Automated perimetry mean deviation (MD) score (≤ 6 months prior to surgery)
- Specular microscopy (≤ 6 months prior to surgery)
- Biometry (e.g., axial length and keratometry) *Note: keratometry data is not required for standalone surgeries*
- Target postoperative refraction, if surgery was a combined cataract/IOL procedure

7.2. Surgical Procedure Data

Where available, the data listed below will be collected in relation to the surgical procedure(s):

- Intraoperative/postoperative steroids used
- CycloPen System serial number(s)
- Cyclodialysis location and relevant anatomic landmarks as assessed by intraoperative gonioscopy
- Number of biotissue implants delivered to the eye via the CycloPen System and material position relative to the cyclodialysis cleft
- Allograft tissue serial number(s), if delivered using the CycloPen System
- Adverse events (noting timing in relationship to CycloPen use)
- The following additional data will also be collected for combined cataract/IOL procedures:
  - Phacoemulsification-related AEs
  - IOL type (accommodative, monofocal – spheric/aspheric, multifocal, toric)

7.3. Postoperative Clinical Assessments

Participants will be evaluated per the investigator's standard practice. Data will be collected for any of the following assessments performed within the 1-month, 6-month, 12-month, 18-month and 24-month postoperative visit windows shown in ***Table 1: Clinical Assessment and Procedure Data to be Collected by Evaluation Time Period***.

- Change in relevant non-ocular medical history
- Change in ocular medical history
- Hypotensive medications used (ocular and systemic medications that may affect IOP)
- Corrected distance visual acuity (CDVA) with refraction

- Slit lamp examination
- Intraocular pressure (IOP)
- Dilated fundus examination* (if macular OCT is performed, images may be collected, if available)
- Automated perimetry MD*
- Gonioscopy (images may be collected, if available)
- Specular microscopy –at approximately 12 and 24 months postoperatively (images may be collected, if available). *Note: If specular microscopy was performed in the early postoperative period but not preoperatively, this data will also be collected*
- Adverse events (AEs)

*If the dilated fundus examination and/or automated perimetry assessments were performed at intervals outside of the 12 month and 24 month visit windows, data from these assessments should be documented via an Unscheduled Visit eCRF.

8. ADVERSE EVENTS

An AE is any untoward medical occurrence, unintended disease or injury, or untoward clinical sign (including an abnormal laboratory finding) or symptom in participants whether or not related to the CycloPen System.

Throughout the study, the investigator should remain alert to possible AEs or untoward findings related to use of the study device. Ocular conditions or diseases present prior to surgery with the CycloPen System that are chronic but stable should be recorded as Ocular History. Changes in any chronic condition or disease consistent with natural disease progression are not considered AEs.

Adverse events must be assessed for severity and relationship to the CycloPen System, and the investigator must take appropriate and necessary therapeutic measures required for AE resolution. Adverse events should be evaluated until resolution or, if the AE is assessed as chronic, until stable. Adverse events that are ongoing at the time of participant study exit should be followed until resolution or stabilization.

8.1. Adverse Device Effects

An Adverse Device Effect (ADE) is an AE related to the use of the CycloPen System. This includes AEs resulting from insufficient or inadequate instructions for use; deployment, operation, or any malfunction of the device; and any AE resulting from use error or intentional misuse of the study device.

An Unanticipated Adverse Device Effect (UADE) is any ADE that seriously affects participant health or safety, or any life-threatening problem or death caused by or associated with the CycloPen System, if that effect, problem, or death was not previously identified in nature,

severity, or degree of incidence in the study protocol.

8.2. Serious Adverse Events

A Serious Adverse Event (SAE) is any untoward medical occurrence that meets any of the following criteria:

- Death
- Is life threatening (places the participant at immediate risk of death from the event as it occurred)
- Requires inpatient hospitalization or prolongation of existing hospitalization
- Results in permanent impairment of a body function or permanent damage to a body structure
- A congenital anomaly or birth defect
- Requires medical or surgical intervention to prevent permanent impairment of a body function or permanent damage to a body structure

Important medical events that may not result in death, be life-threatening, or require hospitalization may be considered serious when, based upon appropriate medical judgment, they may jeopardize the participant and may require medical or surgical intervention to prevent one of the outcomes listed in this definition.

8.2.1. Serious Ocular Adverse Events

Ocular AEs that are considered to be "serious" include, but are not limited to:

- A decrease in best-corrected distance visual acuity (BCDVA) of ≥ 3 lines from the most recent previous measurement of BCDVA
- Severe intraocular inflammation, e.g., hypopyon or 4+ vitritis
- Corneal decompensation
- Severe retinal detachment
- Severe choroidal hemorrhage
- Severe choroidal detachment and aqueous misdirection

8.3. Adverse Event Assessment

Any ocular AE that occurs between the time of use of the CycloPen System in surgery and the participant's exit from the study must be assessed for severity, relationship to the CycloPen System, and event "expectedness."

8.4. Severity

Adverse event severity must be assessed by the investigator using the following definitions:

Mild	Participant is aware of sign or symptom and easily tolerates
Moderate	Participant's discomfort causes interference with normal daily activities
Severe	Participant is unable to perform normal daily activities

8.5. Relationship to Study Device

The relationship of the AE to the CycloPen System must be assessed by the investigator using the following definitions:

Definitely Related	A clear-cut causal relationship; no other possible cause
Probably Related	A causal relationship is likely, although alternative etiologies are possible
Possibly Related	A causal relationship is not definite; alternative etiologies are possible
Not Related	No causal relationship (e.g., there is evidence of alternate etiology, such as concurrent illness or injury)

8.5.1. Expectedness

If the investigator considers the AE to be possibly, probably, or definitely related to the CycloPen System, a determination must also be made regarding expectedness of the event.

Expected AEs associated with IOP-lowering and/or cataract surgical procedures that might reasonably be expected to occur in this study are listed below. These specific examples include, but are not limited to:

Intraoperative Adverse Events

- Posterior capsular rupture
- Anterior capsule tear
- Zonular dialysis
- Inadvertent perforation of sclera
- Hyphema obscuring the surgeon's view
- Inadvertent loss of vitreous
- Choroidal hemorrhage or effusion
- Significant iris injury or trauma
- Significant corneal damage

Postoperative Adverse Events

- Chronic ocular pain, defined as continuous pain documented at 2 visits at least 30 days apart, that is not associated with a pre-existing condition
- Flat anterior chamber requiring anterior chamber reformation
- Shallow chamber with iridocorneal apposition without lens/cornea touch
- Best-corrected visual acuity loss of 2 lines or more measured ≥ 90 days postoperatively
- Clinically significant hypotony, defined as IOP < 6 mmHg that results in maculopathy; flat anterior chamber requiring reformation; corneal folds; choroidal effusion requiring surgical drainage; suprachoroidal hemorrhage; or BCVA loss of ≥ 2 lines
- Macular edema (including cystoid macular edema and diabetic macular edema)
- Hypotonic maculopathy
- Choroidal folds
- Other maculopathy
- Vitreous hemorrhage
- Wound dehiscence, defined as persistent aqueous leak or fistula formation
- Chronic anterior uveitis, defined as inflammation Grade 1+ or worse that persists longer than 90 days, or recurs < 90 days after discontinuation of anti-inflammatory treatment
- Any 2-point worsening in slit lamp examination findings (other than cells and flare) to “severe or “very severe”, which is not associated with a pre-existing condition.
- Endophthalmitis
- Hyphema of > 2 mm present after 1 day postoperatively
- Corneal opacification or corneal decompensation
- Corneal edema (other than mild-to-moderate present < 30 days postoperatively)
- Retinal complications (dialysis, flap tears, retinal detachment, or proliferative vitreoretinopathy)
- Choroidal hemorrhage or choroidal effusion requiring surgical drainage
- Choroidal effusion or detachment with at least a partially hemorrhagic component that obstructs vision or causes pain lasting ≥ 30 days
- IOP ≥ 10 mmHg higher than IOP at the preoperative visit, measured ≥ 30 days postoperatively
- Events resulting in an unplanned ocular surgical reintervention (other than paracentesis to relieve elevated IOP prior to 1 week postoperatively, or Nd:YAG capsulotomy)

- Increase in C:D ratio of ≥ 0.3 units from preoperative
- Confirmed worsening in the visual field MD of ≥ 2.5 dB compared to preoperative

8.6. Adverse Event Documentation and Reporting

The following information should be documented and reported on the relevant eCRF for each ocular AE considered to be possibly, probably, or definitely related to the study device:

- Date of onset
- Date of resolution
- Severity
- Nature of the event (intermittent, continuous)
- Action taken (none, medical and/or surgical)
- Relationship to study device/surgical procedure
- Seriousness
- Expectedness

Any medication or other intervention necessary for the treatment of the AE should be recorded on the appropriate eCRF. If the same type of AE occurs multiple times, each event should be recorded separately. If an AE is considered Serious, the following information will be collected on an additional eCRF:

- Date site became aware of event
- Expectedness
- SAE classification

8.6.1. Expedited Adverse Event Reporting

User facilities are required to report any AE that reasonably suggests that the CycloPen System caused or contributed to a death or serious injury to FDA and to Iantrek within 10 days of becoming aware of the event. *Note: If the reportable AE is related to allograft scleral tissue or other accessory material delivered by the CycloPen, the distributor of that product should be promptly notified.*

Reports to FDA must be made using MedWatch Form 3500A. Reports to Iantrek should be made by completing the SAE eCRF. This information may be supplemented with other medical records evaluating the participant’s condition, etc. as applicable for the event.

9. STATISTICAL CONSIDERATIONS

9.1. Sample Size

As there are no formal hypotheses for this study, the sample size is based on achieving a maximum confidence interval (CI) width of approximately 20% (Estimate ± 10%). As described in the ***CREST Registry Statistical Analysis Plan*** (SAP), a sample of 70 analyzable eyes in the smallest cohort provides the required CI width.

9.2. Analysis Populations

9.2.1. Safety Population

The Safety Population (SAF) includes all eyes with an attempted surgery with the CycloPen System. The SAF will be used as the primary analysis population for all safety analyses.

9.2.2. Treated-Eye Population

The Treated-Eye Population (TRT) includes all eyes that underwent successful surgery with the CycloPen System and meet the clinical criteria listed in ***Table 2: TRT Population Clinical Criteria***, have 12-month and/or 24-month follow-up, and no major protocol deviations. The TRT will be used for all effectiveness analyses.

Table 2: TRT Population Clinical Criteria

All eyes included in the TRT population must meet the following clinical criteria:

- Diagnosis of OAG
- CycloPen System procedure with delivery of allograft tissue for reinforced cyclodialysis absent any additional concomitant IOP-lowering adjunct intervention, other than cataract surgery for eyes in Cohort 1
- Absent any other IOP-lowering procedure ≤ 12 weeks prior to CycloPen System surgery
- Axial length ≤ 26.0 mm
- Absent any investigational, or non-standard of care procedures and/or treatments ≤ 3 months prior to CycloPen System surgery

Note that the TRT population is analogous to the "per-protocol" population in a randomized clinical trial. For each outcome, all analyzable participants will be included in the analysis. In particular, outcomes 1, 2, 3, and 7 will use all participants with 12-month outcomes; outcomes 4, 5, 6, and 8 will use all participants with 24-month outcomes; outcomes 9, 10, 11, and 12 will use all participants with both 12- and 24-month outcomes.

9.3. Data Analysis Conventions

Data will be summarized in tables, with underlying source data provided in listings, ordered by participant, and visit (if applicable).

The unit of analysis will be eyes. Both eyes, when available, will be displayed in summary tabulations and analyzed for all effectiveness and safety outcomes. All effectiveness analyses will use methods that account for correlation between eyes.

9.4. Baseline Definition

For all outcomes, baseline is defined as the last measurement recorded prior to surgery. Change from baseline will be calculated as Post-Baseline Measure – Baseline Measure. Percent decrease from baseline will be calculated as 100% × (Baseline Measure – Post-Baseline Measure) / Baseline Measure.

9.5. Descriptive Statistics

Descriptive statistics for demographics, ocular medical history, and biometry at the baseline visit will be tabulated. Descriptive statistics for IOP, ocular medications, and CDVA will be tabulated at baseline, 1, 6, 12, 18, and 24 months. All safety outcomes will be tabulated, and descriptive statistics calculated by cohort and visit.

For continuous measures, descriptive summary statistics will include the number of observations, mean, standard deviation, median, minimum, maximum. Minima and maxima will be reported with the same precision as the raw values; means and medians will be presented to 1 additional decimal place than reported in the raw values. Standard deviations will be presented to 2 additional decimal places than reported in the raw values. For categorical measures, the number and percentage of cases for each condition (e.g., IOP ≤ 18 mmHg), along with the 95% Wilson score confidence interval (that is, not accounting for intraclass correlation) will be reported. The number of eyes without results will be omitted from the numerator and denominator of such calculations. All percentages will be rounded to 1 decimal place (i.e., XX.X%).

9.6. Effectiveness Outcome Analysis

Effectiveness outcomes to be analyzed are listed in order of clinical importance in ***Section 1 Effectiveness Outcomes***. As there are no pre-specified hypotheses to be tested, results will consist of point estimates and CIs for each effectiveness outcome. All effectiveness outcomes assessed will be based on IOP and/or ocular hypotensive medication use. All effectiveness analyses will be carried out separately by cohort. Analyses will be performed at the 12- and/or 24-month time points, as described for each outcome in ***Section 3.1 – Effectiveness Outcomes***.

For binary variables, the proportion of eyes that meet the success definition will be calculated. For integer variables (medication counts), the median change from baseline in the number of medications will be calculated. For continuous variables (IOP change), the mean of the per-eye change from baseline will be calculated.

As participants may have undergone surgery with the CycloPen System in both eyes, dependence between eyes is likely. Thus, for all outcomes, 95% confidence intervals for the estimate will be calculated using a participant-level clustered bootstrap.

9.6.1. Exploratory Analyses

For exploratory purposes, effectiveness analyses described in ***Section 10*** may be reprised on differing subsets of the TRT population.

9.7. Safety Outcome Analysis

Safety will be assessed in a pooled cohort and separately in the 2 key participant cohorts below:

- Cohort 1: CycloPen procedure combined with cataract extraction/intraocular lens (IOL) implantation, and
- Cohort 2: CycloPen procedure as a standalone surgical intervention.

Safety data will be tabulated showing the incidence per eye for ocular AEs and other safety measures, with number and percentage of exposed eyes. Safety outcomes will include measures taken after surgery, with particular attention to CycloPen System-related AEs. Safety will be summarized over all follow-up as well as by follow-up periods of interest.

9.7.1. Visual Field Change

Available visual field mean deviation (MD) will be summarized with N, mean, standard deviation, minima, median, and maxima at baseline and postoperative months 12 and 24.

9.7.2. Endothelial Cell Loss

For eyes with analyzable endothelial cell count (ECC) results, data will be summarized at baseline, and postoperative months 12 and 24 with N, mean, standard deviation, minimum, and maximum.

9.8. Multiplicity and Missing Data

Only available data will be used, with no imputation of missing data. No adjustment for multiplicity will be performed.

Additional details regarding the planned summaries and analyses of study data are included in the ***CREST Registry Study Statistical Analysis Plan***.

10. SUPPORTING DOCUMENTATION AND OPERATIONAL CONSIDERATIONS

10.1. Data Management

Prior to initiation of data collection, a data management plan will be created, which describes all functions, processes, and specifications for data collection, cleaning, and validation. Study eCRFs will include programmable edits to obtain immediate feedback if data is missing, out of range, illogical, or potentially erroneous. Concurrent manual data review will be performed based on parameters dictated by the plan.

10.2. Data Capture, Entry, and Quality Control

Study data will be collected and entered directly onto eCRFs maintained in the CREST Registry electronic data capture (EDC) system by personnel at the site or provided by the Sponsor. Participating sites will have access to data entered for participants enrolled at their individual site. Designated data entry personnel will be trained to use the EDC system, including eCRF completion guidelines and data coding recommendations.

After entry of data into the EDC system, computerized data validation checks will be applied and queries pertaining to data omissions and discrepancies will be directed to data entry personnel for resolution. Data entry personnel will update the database as appropriate to resolve queries generated. Changes or corrections to eCRFs will be documented via an automated audit trail, with an adequate explanation for any changes made.

10.3. Data Archiving

Archived versions of the database will be saved by Iantrek consistent with ICH GCP Guidelines.

10.4. Records Retention

The study site will retain all records related to the study in compliance with ICH GCP Guidelines.

10.5. Quality Assurance

The study database will be maintained by Iantrek in a logically secure system meeting the standards of 21 CFR Part 11 – Electronic Records; Electronic Signatures.

Due to the nature of the study, it is anticipated that most quality controls will be implemented through edit checks and data queries issued through the EDC system; however, Iantrek may perform periodic visits to study sites to verify information on the study eCRFs against source documents to confirm data capture completeness, accuracy, and logical consistency.

10.6. Protocol Amendments

Modifications to the approved protocol are only possible with an approved protocol amendment and the agreement of all responsible persons. The Institutional Review Board (IRB) or Ethics Committee (EC) must be informed of any protocol amendments and asked to evaluate the

nature of amendment. Records of the IRB/EC review and decision regarding the amendment(s) should be maintained.

10.7. Publication Policy

Any publication of results from this study must be consistent with the Uniform Requirements for Manuscripts Submitted to Biomedical Journals: Writing and Editing for Biomedical Publication of the International Committee of Medical Journal Editors (ICMJE), updated April 10, 2020.

Study investigators must submit any manuscripts, posters, abstracts, or other intended publications for Iantrek's review at least 60 days prior to planned submission for publication and Iantrek will promptly review the proposed manuscript, providing approval/disapproval. In the event the proposed publication is disapproved, Iantrek will work with the investigator to address associated concerns. In no event shall any manuscript be submitted for publication without the prior written approval of Iantrek.

11. ETHICAL AND REGULATORY CONSIDERATIONS

The study will be conducted in compliance with the 21 CFR Part 50 – Protection of Human Patients and/or Part 56 – Institutional Review Boards; the ICH GCP guidelines as they apply to post-market observational studies; the Declaration of Helsinki and its amendments; and the Health Insurance Portability and Accountability Act (HIPAA) of 1996.

11.1. Risks and Benefits of Participation

Patient participation in this study is considered to involve minimal risk. The primary potential risk is inadvertent release of personal medical data; however, safeguards will be enacted during the conduct of this study to minimize the likelihood of a breach of patient confidentiality.

The primary purpose of this study is to generate additional post-market evidence regarding patient outcomes after use of the CycloPen System for IOP-lowering surgical procedures. Patients that participate in the study will receive no direct benefit, but the knowledge gained regarding treatment outcomes is of value to public health.

11.2. Patient Information and Informed Consent

The IRB/EC-approved study informed consent form (ICF) must be used for administration of participant informed consent to enroll in this study. After the clinical decision to perform IOP-lowering surgery using the CycloPen System and evaluation of the participant's eligibility for the study, the investigator or designee will explain the study purpose, procedures and responsibilities to the potential participant and provide sufficient opportunity to ask questions while allowing adequate time for consideration of the information provided. The ICF must be signed by the participant (or his/her legally authorized representative) after the surgical procedure before enrollment and participation in the study.

The participant's medical record should document that written informed consent was obtained prior to study participation. A copy of the signed ICF must be provided to the participant (or his/her legally authorized representative). All signed and dated ICFs must be maintained in each participant's study file.

The study-specific ICF and HIPAA Authorization for Release of Information template will be provided to sites to facilitate their IRB/EC submission process. If changes are made to procedures outlined in the ICF or if new information becomes available that might affect participant willingness to continue study participation, the ICF should be revised and reviewed by the IRB/EC. Upon approval of the revised ICF, the IRB/EC will provide instructions for participant re-consent and the investigator is expected to adhere to these IRB/EC requirements. Documentation of appropriate informed consent is subject to audit.

11.3. Participant Confidentiality

Each participant will be assigned a unique identifier upon study enrollment. This identifier will be used in place of the participant's name for the purpose of data analysis and reporting. The participant's medical record number or other local reference identifiers will not be collected within the database.

Site and Iantrek personnel will ensure protection of participant personal data and will not include participant names on any forms, reports, publications, or in any other disclosures, except where required by law. Participants will be informed about data handling procedures and asked for their consent to provide data for the study. Data protection and privacy regulations will be observed in capturing, forwarding, processing, and storing patient data. Study records may be made available for review as required by governing regulatory authorities or reviewing IRB(s)/EC(s); however, to the extent possible, participant identities will not be disclosed.

11.4. Institutional Review

Consistent with local regulations and prior to enrollment of participants at a given site, the study protocol will be submitted together with its associated documents (e.g., ICF) to the responsible central and/or local IRB/EC for its review, as required. Participant enrollment will not start at any site before the study has obtained written confirmation of a favorable opinion/approval from the relevant central or local IRB/EC. The IRB/EC will be asked to provide documentation of the date of the meeting at which the favorable opinion/approval was given that clearly identifies the study, the protocol version, and the ICF version reviewed.

Before implementation of any substantial changes to the protocol, protocol amendments will also be submitted to the relevant IRB/EC in a manner consistent with local regulations. Pertinent safety information will be submitted to the relevant IRB/EC during the course of the study in accordance with local requirements. It is the responsibility of the site to have prospective approval of the study protocol, protocol amendments, and informed consent forms, and other relevant documents, if applicable, from their local IRB/EC and provide documentation of approval to the study.

12. REFERENCES

1. Friedman DS, Wolfs RC, O'Colmain BJ, et al. Prevalence of open-angle glaucoma among adults in the United States. Arch Ophthalmol 2004;122:532-8.
2. Congdon N, O'Colmain B, Klaver CC, et al. Causes and prevalence of visual impairment among adults in the United States. Arch Ophthalmol 2004;122:477-85.
3. Sommer A, Tielsch JM, Katz J, et al. Racial differences in the cause-specific prevalence of blindness in east Baltimore. N Engl J Med 1991;325:1412-7.
4. Primary Open Angle Glaucoma. Preferred Practice Patterns, American Academy of Ophthalmology.http://one.aao.org/CE/PracticeGuidelines/PPP_Content.aspx?cid=a5a59e02-450b-4d50-8091-b2dd21ef1ff2#references (Accessed 11-1-08).
5. Jay JL, Allan D. The benefit of early trabeculectomy versus conventional management in primary open-angle glaucoma relative to severity of disease. Eye 1989;3:528-35.
6. Migdal C, Gregory W, Hitchings R. Long term functional outcome after early surgery compared with laser and medicine in open-angle glaucoma. Ophthalmology 1994;101:1651-7.
7. The Glaucoma Laser Trial (GLT). 2. Results of argon laser trabeculoplasty versus topical medicines. The Glaucoma Laser Trial Research Group. Ophthalmology 1990;97:1403-13.
8. The Glaucoma Laser Trial (GLT) and Glaucoma Laser Trial Follow-up Study: 7. Results. Am J Ophthalmol 1995;120:718-31.
9. Kass MA, Heuer DK, Higginbotham EJ, et al for the Ocular Hypertension Treatment Study Group. The Ocular Hypertension Treatment Study. A randomized trial determines that topical ocular hypotensive medication delays or prevents the onset of primary open-angle glaucoma. Arch Ophthalmol 2002;120:701-13.
10. Gordon MO, Kass MA, for the Ocular Hypertension Treatment Study Group. The Ocular Hypertension Treatment Study. Design and baseline description of the participants. Arch Ophthalmol 1999:573-83.
11. Collaborative Normal-Tension Study Group. Comparison of glaucomatous progression between untreated patients with normal-tension glaucoma and patients with therapeutically reduced intraocular pressures. Am J Ophthalmol 1998;126:487-97.
12. Heijl A, Leske MC, Bengtsson B, et al for the Early Manifest Glaucoma Trial Group. Reduction of intraocular pressure and glaucoma progression. Results from the Early Manifest Glaucoma Trial. Arch Ophthalmol 2002;120:1268-79.

13. Leske MC, Heijl A, Hussein M, et al for the Early Manifest Glaucoma Trial Group. Factors for glaucoma progression and the effect of treatment. The Early Manifest Glaucoma Trial. Arch Ophthalmol 2003;121:48-56.
14. Lichter PR, Musch DC, Gillespie BW, et al and the CIGTS Study Group. Interim clinical outcomes in the Collaborative Initial Glaucoma Treatment Study comparing initial treatment randomized to medications or surgery. Ophthalmology 2001;108:1943-53.
15. The AGIS Investigators. The Advanced Glaucoma Intervention Study (AGIS): 7. The relationship between control of intraocular pressure and visual field deterioration. Am J Ophthalmol 2000;130:429-40.
16. The AGIS Investigators. The Advanced Glaucoma Intervention Study (AGIS): 13. Comparison of treatment outcomes within race. Ten-year results. Ophthalmology 2004;111:651-64.
17. Miglior S, Pfeiffer N, Zeyen T et al for the European Glaucoma Prevention Study Group. Results of the European Glaucoma Prevention Study. Ophthalmology 2005;112:366-75.
18. Miglior S, Zeyen T, Pfeiffer N, et al for the European Glaucoma Prevention Study Group. The European Glaucoma Prevention Study design and baseline description of the participants. Ophthalmology 2002;109:1612-21.
19. The Advanced Glaucoma Intervention Study (AGIS): 7. The relationship between control of intraocular pressure and visual field deterioration. The AGIS Investigators. Am J Ophthalmol 2000;130:429-40.
20. Ritch R, Shields MB, Krupin T. *The Glaucomas*. St. Louis: Mosby, 1996; 337 – 343).
21. Toris CB. Extravascular albumin concentration of the uvea. *Invest Ophthalmol Vis Sci* 1987; 28: 477.
22. Suguruo K, Toris CB, Pederson JE. Uveoscleral outflow following cyclodialysis in the monkey eye using a fluorescent tracer. *Invest Ophthalmol Vis Sci* 1985: 26, 810.

13. INVESTIGATOR ATTESTATION

AN OBSERVATIONAL REGISTRY STUDY OF SAFETY AND EFFECTIVENESS OUTCOMES THROUGH 24 MONTHS POSTOPERATIVELY FOLLOWING CYCLOPEN™ MICRO-INTERVENTIONAL CYCLODIALYSIS SYSTEM PROCEDURES IN PATIENTS WITH OPEN ANGLE GLAUCOMA (THE CREST STUDY)

SPONSOR:

IANTREK, INC
151 E. POST RD, SUITE 111
WHITE PLAINS, NY 10601

PHONE: [redacted]

I have read this protocol in its entirety. I agree to:

- Implement and conduct this study in compliance with this study protocol; conditions of approval imposed by my reviewing IRB/EC; Good Clinical Practice (GCP); and any other applicable laws and regulations.
- Maintain all study-related information supplied by Iantrek in a confidential manner.

Protocol Amendment

Modification of the study protocol is prohibited without prior written agreement in the form of a protocol amendment. All amendments will be created by Iantrek and must be approved by the reviewing IRB/EC prior to implementation at your site, except when required to mitigate immediate safety risks or when the changes involve only logistical or administrative revisions.

____________________	____________________	__________
Investigator Name	Signature	Date
